# Subjective and objective demands on different types of differential stress inventory

**DOI:** 10.1007/s00420-020-01632-4

**Published:** 2021-01-13

**Authors:** Håvard R. Karlsen, Irina Böckelmann, Beatrice Thielmann

**Affiliations:** 1 Department of Psychology, Norwegian University of Science and Technology, Trondheim, Norway; 2 Institute of Occupational Medicine, Medical Faculty, Otto von Guericke University, Leipziger Straße 44 (Building 20), 39120 Magdeburg, Germany

**Keywords:** Burnout, Heart rate variability, Stress, Maslach burnout inventory, Differential stress inventory

## Abstract

**Purpose:**

To validate the differential stress inventory (DSI) by evaluating the objective and subjective stress differences in the five DSI types in the occupational setting.

**Methods:**

A total of 119 German participants working as medical assistants (*n* = 40) or in a bank (*n* = 79) were recruited. They completed the Maslach Burnout Inventory–General Survey, the DSI, and wore ECG measuring devices for 24 h to measure heart rate variability. The DSI was used to group people into one of five types according to how they perceived and coped with stress: normal, overstressed, stress-resistant, low stress/high coping, or high stress/high coping.

**Results:**

The overstressed type had significantly more burnout symptoms than the other types. The high stress/high coping type also had more symptoms of emotional exhaustion and total burnout compared to the other types, while the low stress/high coping and the stress-resistant types generally had the lowest levels of burnout. There were no differences on the HRV parameters among the DSI types.

**Conclusion:**

Categorising people into types like in the DSI can help make workers aware of unhealthy stress and coping patterns before they turn into more severe pathology. Proper application and targeted preventive measures can save the individual’s health and the company’s budget. While the DSI picked up on differences in burnout symptoms as a long-term consequence of stress, there is evidence that it cannot pick up on short-term stress or physical stress as measured by HRV from the 24 h recording.

## Introduction

The digitalisation of the workplace does not automatically cause more healthy work. There are not only advantages, but also disadvantages, such as the increase in mental stress caused by information overload or constant availability. As a result, work and private environment bleed into each other (Minow and Swart 2019). For example, writing and checking e-mails in free time lead to an increased feeling of overload (Minow and Swart 2019). An unbalanced relationship between effort and reward can lead to poor performance, which can result in mental illness. Studies have shown an increased risk of depression or burnout for employees with an imbalance of effort and reward (Rugulies et al. 2013; Looseley et al. 2019). The cost of mental illness is forecast to rise worldwide from $2.5 trillion in 2010 to $6.0 trillion in 2030 (excluding costs for secondary diseases of mental illness, such as cardiovascular disease or diabetes) (Zylka-Menhorn 2011).

Thus, mental illnesses remain of interest for occupational medicine as well as for society and health economics. The subjective and objective measurement of mental stress is important in occupational medicine consultations (Böckelmann and Seibt 2011). Work-related stress can be described as a reaction mechanism, which occurs when employees are faced with work demands that do not conform to their knowledge, skills, or abilities and that challenge their ability to cope. Knowledge about individual stress and coping with stress is important for personal well-being and professional success (Lefèvre and Kubinger 2004). The individual stress experience or behaviour can be investigated by means of the Differential Stress Inventory (DSI). It measures stress triggers, manifestations, and stabilisation or coping strategies (Lefèvre and Kubinger 2004). This inventory was constructed based on the concept of performance anxiety diagnostics (“Differential Anxiety Inventory”, DAI) and provides information about the personal handling of stress (Rost and Schermer 1987). Knowledge of work-related and individual resources or personality traits is helpful for preventive health support programs in the workplace (Melzer and Hubrich 2014; Buck et al. 2019). However, there is little published research on the DSI.

For the subjective personal assessment of long-term negative consequences of stress or health impairments, the Maslach Burnout Inventory-General Survey (MBI-GS, (Schaufeli et al. 1996)) was used. Heart rate variability (HRV) was used to provide an objective assessment of mental stress and burnout (Järvelin-Pasanen et al. 2018; Lo et al. 2020). It is a very sensitive indicator, with decreased HRV indicating psychological stress or fatigue and increased HRV indicating relaxation and recovery. Studies have shown a low heart rate variability in patients with burnout (Lennartsson et al. 2016; Lo et al. 2020).

The aim of the study was to validate the DSI questionnaire by evaluating the subjective and objective strains on the different types of people according to the DSI. We hypothesized that there would be differences among the DSI stress-handling types in terms of their burnout manifestations and heart rate variability.

## Materials and methods

### Subjects

101 women (84.9%) and 18 men (15.1%) (*n* = 119; total age 43.2 ± 9.61 years) were included in this survey. The subjects were divided into two occupational groups with psychomental stress at workplace [79 (66.4%) bank employees; 40 (33.6%) medical assistants]. The data are taken from major studies on “Mental health of bank employees/medical assistants”. The groups were selected as all of their work involved personal contact and social responsibility. The profession of medical assistance belongs more to the administrative professions than to the nursing professions. Twenty percent of the bank employees worked in a management position, 96% worked 30–40 h per week, and 97% had permanent contracts. Workplace-related stress factors identified among bank employees were mainly increased data maintenance, increased demands on PC skills, poor communication structures, hierarchical performance pressure, and pressure to meet targets. Eighty-two percent of the medical assistance were employed on a permanent contract and 95% worked 30–40 h per week without shift work. In the case of medical assistance, the main stress factors were patient-related (e.g., number, behaviour, and expectations), increased data maintenance, and increased demands on PC skills. Thus, the populations that form the basis of generalization were German workers in the professions of bank employees and medical assistants.

The individual employers of the stated occupational groups were contacted in advance. Employees were informed about the planned studies by means of flyers, which were given out during health days or sent by e-mail via the administration. Participation in the study was voluntary. Heart disease (e.g., cardiac arrhythmia), diabetes mellitus, and drugs (antiarrhythmic agents and psychotropic drugs) were exclusion criteria. Data were collected from both groups between 2013 and 2014.

The Otto von Guericke University in Magdeburg, Germany (register no. 63/13, 67/13) approved the ethical aspects of the study. The study complied with the guidelines of Declaration of Helsinki.

### Procedure

Standardised questionnaires were used for data collection. The differential stress inventory (DSI) according to (Lefèvre and Kubinger 2004) was used to determine how individuals manage stress. Based on the responses to the DSI items, the subjects were grouped into five different types of Differential Stress Inventory. We looked for differences between the five types of DSI in the subjective long-term stress response, i.e., between the scales and total risk of burnout using the Maslach Burnout Inventory-General Survey (MBI-GS) based on (Maslach and Jackson 1986) and (Schaufeli et al. 1996). To measure strain objectively, we used heart rate variability (HRV) according to the occupational medicine guidelines of Sammito et al. (2015).

The differential stress inventory (DSI) (Lefèvre and Kubinger 2004) examines statements on four stress-related topics (stress triggers, stress manifestation, coping, and stress stabilisation). It distinguishes between the physiological, cognitive, and emotional levels of manifestation. The stress triggers comprise three areas: existential worries, problems arising from interaction with other people, and stressful day-to-day situations. The DSI only records stressful events without positive aspects of stress or life events taken into account. It separates between problem-related (instrumental) and emotional (palliative) coping strategies such as positive emotions and cognition as well as active action against the cause of stress. Stress stabilisation means conditions that maintain or foster stress. Stress stabilisation can occur internally through, for instance, brooding and rumination, or externally through, for instance, recognition by others of one’s accomplishments in the face of the stress.

The following types of DSI are defined:

- DSI type I = normal: all variables within the normal range, average levels of stress with successful coping
- DSI type II = overstressed: above-average stress from everyday life and existential fears, there are problems due to interactions with other people and a high degree of stress triggers, there is instrumental and problem-related coping, but also pronounced external enhancers, and possible chronification.
- DSI type III = stress resistant: lower exposure to stress triggers such as everyday life, existential worries, and interaction with other people, but hardly any recognition of palliative coping.
- DSI type IV = low stress/high coping (LSHC): below average level of stress triggers, hardly any physical or emotional–cognitive discomfort, but above-average palliative coping
- DSI type V = high stress/high coping (HSHC): above-average stress from work and private interaction, but also above-average palliative coping.

The five types of DSI were formed according to the procedure described in the test manual of DSI (Lefèvre and Kubinger 2004). A profile is created which has at least 50% expression of one type and not more than 35% in any other type. Combinations of patterns are not taken into account, as these were not recorded in the manual by the authors.

The Maslach Burnout Inventory-General Survey (MBI-GS) is used to evaluate the burnout risk of the participants (Maslach and Jackson 1986; Schaufeli et al. 1996). The questionnaire include 16 items, which can be combined into three burnout dimensions: (1) emotional exhaustion (EE), (2) cynicism (CY), and (3) professional efficacy (PE). The participants indicate how often they feel each item on a seven-level scale from accomplishment 0 = “never” to 6 = “daily” in the past. During evaluation, the mean value is first calculated for each dimension. The burnout dimensions were combined into a total burnout score according to Kalimo et al. (2003). In the analyses, we focus on the burnout scales, as they more accurately convey the dimensionality of burnout.

The heart rate variability (HRV) gives information about dynamics and mechanisms of the regulation of the cardiovascular system. The quality criteria of the AWMF-s2k guidelines (Sammito et al. 2015), of the Task Force of the European Society of Cardiology and the North American Society of Pacing and Electrophysiology (Task Force of the European Society of Cardiology and the North American Society of Pacing and Electrophysiology 1996) and of the Position Statement by the e-Cardiology ESC Working Group and the European Heart Rhythm Association Co-Endorsed by the Asia Pacific Heart Rhythm Society (Sassi et al. 2015) are taken into account in both the recording and the evaluation of the NN intervals.

The participants used a mobile two-channel ECG device from Schiller AG (model MT-101) from Switzerland over 24 h to record the NN intervals. The recorded NN intervals were transferred to the Medilog DARWIN software taking into account a sampling frequency of 1000 Hz. The program Kubios HRV Premium (Kubios, Kuopio, Finland) was used to calculate the HRV parameters in the time domain, frequency domain, and with non-linear methods (Tarvainen et al. 2019). HRV is influenced by a number of physiological factors such as various diseases. Knowledge of the confounders is important in the analysis and evaluation of HRV (Sammito and Böckelmann 2016). The test persons were instructed to follow their usual daily routines and conducted an activity log. This was used in the evaluation of the HRV Data.

### Statistical analyses

The ECG data was imported into Kubios HRV, where it was analysed to produce heart rate variability data. All analyses were performed in R software version 3.6.3. Summary statistics were computed for all variables. For the continuous variables, we report the mean, standard deviation, 95% confidence interval, median, minimum, and maximum values, while for the categorical variable, the frequency and percentage in each group is reported. One-way ANOVAs were calculated to test for differences between the DSI groups on the continuous variables. We used Levene’s test to check for homoskedasticity and produced quantile–quantile plots to check for normality. In the presence of heteroskedasticity, we used Welch’s *F* test instead of the normal *F* test. For categorical variables, Fisher’s exact test was used to test for significant differences between the DSI groups, due to the small expected count in some cells. Equivalence among the DSI groups on the background variables of age, height, and weight were tested using the confidence interval method (Rogers et al. 1993; Rusticus and Lovato 2011). We set an equivalence interval at ± 0.5 SD based on the finding that 0.5 of the SD is a relatively consistent minimally important difference in health outcomes (Norman et al. 2003). We concluded with equivalence if the 90% confidence interval was contained within the equivalence interval. We tested for any dependency between the DSI groups and, respectively, work groups, gender, age, height, and weight. As there was no proof of any dependencies, we proceeded to focus solely on potential differences on the background, MBI and HRV variables among the DSI groups. The level of significance was set at 0.05.

## Results

### Analyses of background data

Descriptive statistics for background variables of the test persons are shown in Table 1. The 90% confidence interval of the mean difference between DSI groups on age, height, and weight was not contained within any of the respective equivalence intervals (see Table 2). The analyses thus suggested that the groups were not equivalent on any of these variables. The overstressed group was the oldest, followed by the stress resistant, normal, high stress–high coping, and low stress–low coping groups. The low stress–high coping group was the tallest, followed by the stress resistant, normal, overstressed, and high stress–high coping groups. The heaviest groups were, in descending order: low stress–high coping, overstressed, stress resistant, normal, and high stress–high coping. Potential relationships between the DSI groups and gender and workgroup were investigated by Fisher’s exact test. There were no significant relationships between the DSI groups and gender (*p* = 0.722) or workgroup (*p* = 0.841).

**Table 1:** Descriptive statistics for background variables

	Differential stress inventory groups						
	Total sample	Normal	Overstressed	Stress resistant	Low stress/high coping	High stress/high coping	DSI groups’ differences
	(*n* = 119)	(*n* = 58)	(*n* = 11)	(*n* = 21)	(*n*= 17)	(*n* = 12)	
Gender							
Male	18 (15.1%)	7 (12.1%)	1 (9.1%)	4 (19.0%)	4 (23.5%)	2 (16.7%)	*p* = .722
Female	101 (84.9%)	51 (87.9%)	10 (90.9%)	17 (81.0%)	13 (76.5%)	10 (83.3%)	–
Workgroup							
Bank	79 (66.4%)	37 (63.8%)	7 (63.6%)	13 (61.9%)	13 (76.5%)	9 (75.0%)	*p* = .841
MFA	40 (33.6%)	21 (36.2%)	4 (36.4%)	8 (38.1%)	4 (23.5%)	3 (25.0%)	–
Age							
Mean ± SD	43.19 ± 9.61	43.66 ± 9.93	45.38 ± 6.93	44.15 ± 10.50	40.23 ± 10.07	41.42 ± 8.03	–
CI	(41.46, 44.92)	(41.11, 46.22)	(41.28, 49.48)	(39.66, 48.64)	(35.44, 45.01)	(36.87, 45.96)	–
Median	43	43	45	45	39	42	–
Minimum	22, 11	22, 11	36, 8	23	26	27	–
Maximum	61, 5	61, 5	59	59	61	52, 1	–
Height (cm)							
Mean ± SD	168.71 ± 8.63	168.19 ± 7.31	167.27 ± 7.48	169.57 ± 8.39	173.56 ± 9.87	164.58 ± 12.06	–
CI	(167.16, 170.27)	(166.31, 170.07)	(162.85, 171.70)	(165.98, 173.16)	(168.73, 178.40)	(157.76, 171.41)	–
Median	168	168	165	168	174, 5	160, 5	–
Minimum	145	153	156	157	155	145	–
Maximum	193	190	184	191	193	187	–
Weight (kg)							
Mean ± SD	72.91 ± 15.23	72.63 ± 15.23	74.98 ± 16.39	73.19 ± 15.69	77.06 ± 13.18	66.33 ± 16.15	–
CI	(70.15, 75.67)	(68.68, 76.58)	(65.30, 84.66)	(66.47, 79.90)	(70.60, 83.52)	(57.19, 75.47)	–
Median	70	70	78	68	74, 5	61, 5	–
Minimum	42	44	55	52	56	42	–
Maximum	110	110	107	100	102	104	–

For continuous variables, mean, standard deviation, 95% confidence interval, median, minimum, and maximum values are reported. For categorical variables, n and % are reported, along with results from Fisher's exact test. For tests of the equivalence of groups on age, height, and weight, see Table 2

**Table 2:** Group differences, 90% confidence intervals, and equivalence tests

Comparisons	Age			Height			Weight		
	MD	CI_LL_	CI_UL_	MD	CI_LL_	CI_UL_	MD	CI_LL_	CI_UL_
Normal–LSHC	− 3.43	− 10.63	3.76	5.37	− 1.63	12.38	4.43	− 5.58	14.45
Normal–overstressed	1.72	− 4.82	8.26	− 0.92	− 7.61	5.77	2.35	− 12.24	16.95
Normal–resistant	0.49	− 6.27	7.25	1.38	− 3.94	6.70	0.56	− 9.63	10.74
Normal–HSHC	− 2.24	− 9.31	4.82	− 3.61	− 13.55	6.34	− 6.30	− 20.01	7.42
LSHC–overstressed	5.15	− 3.20	13.51	− 6.29	− 15.01	2.43	− 2.08	− 17.87	13.70
LSHC–resistant	3.92	− 4.65	12.50	− 3.99	− 11.93	3.95	− 3.88	− 16.06	8.30
LSHC–HSHC	1.19	− 7.55	9.93	− 8.98	− 20.22	2.26	− 10.73	− 25.77	4.31
Overstressed–resistant	− 1.23	− 9.25	6.80	2.30	− 5.32	9.91	− 1.80	− 17.70	14.11
Overstressed–HSHC	− 3.97	− 12.19	4.26	− 2.69	− 13.71	8.34	− 8.65	− 26.55	9.25
Resistant–HSHC	− 2.74	− 11.17	5.70	− 4.99	− 15.51	5.53	− 6.85	− 22.02	8.31

Equivalence interval: age = (− 4.81, 4.81), height =(− 4.32, 4.32), weight = (− 7.62, 7.62)

*LSHC*  low stress, high coping, *HSHC*  high stress, high coping, *MD*  mean difference, *CI * confidence interval, *LL*  lower limit, *UL*  upper limit

### Analyses of MBI

**Fig. 1:**
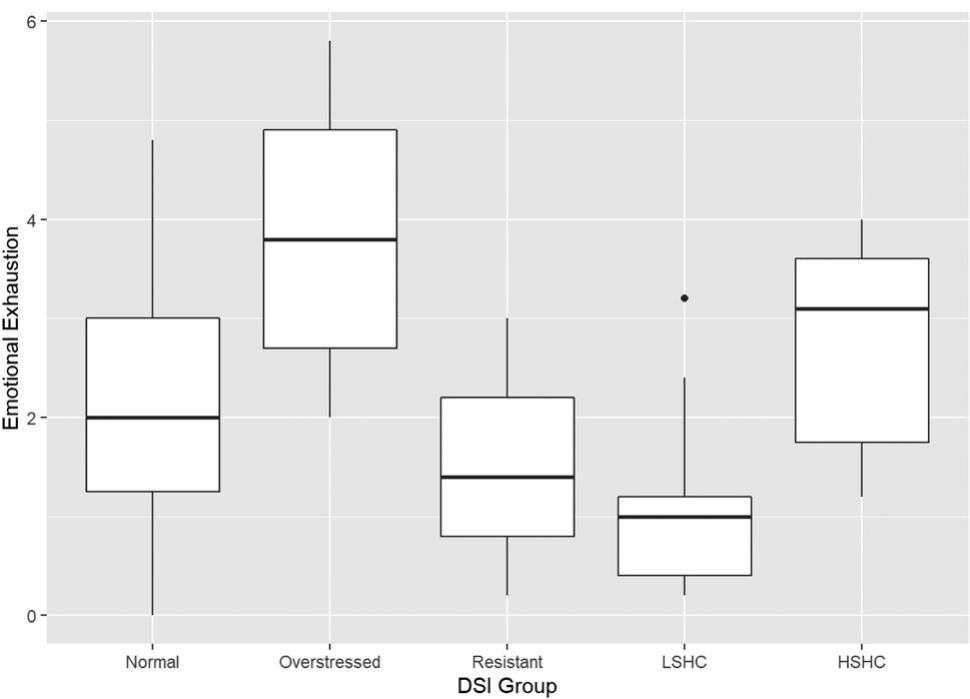
Boxplot of emotional exhaustion in the DSI groups. (*LSHC* low stress – high coping,* HSHC* high stress – high coping)

**Fig. 2:**
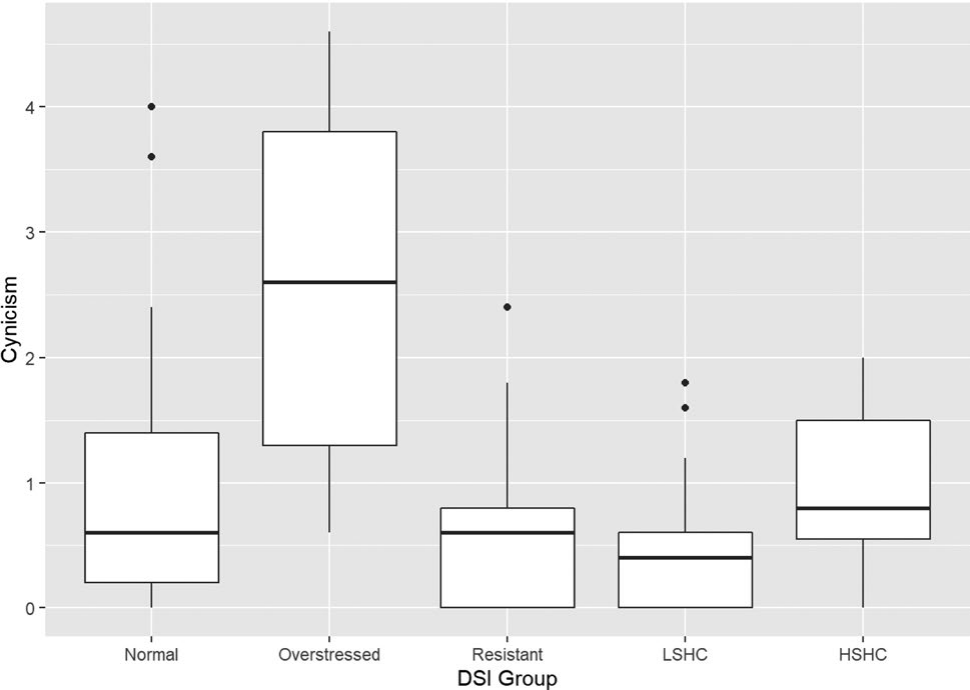
Boxplot of cynicism in the DSI groups. (*LSHC* low stress – high coping,* HSHC* high stress – high coping)

**Fig. 3:**
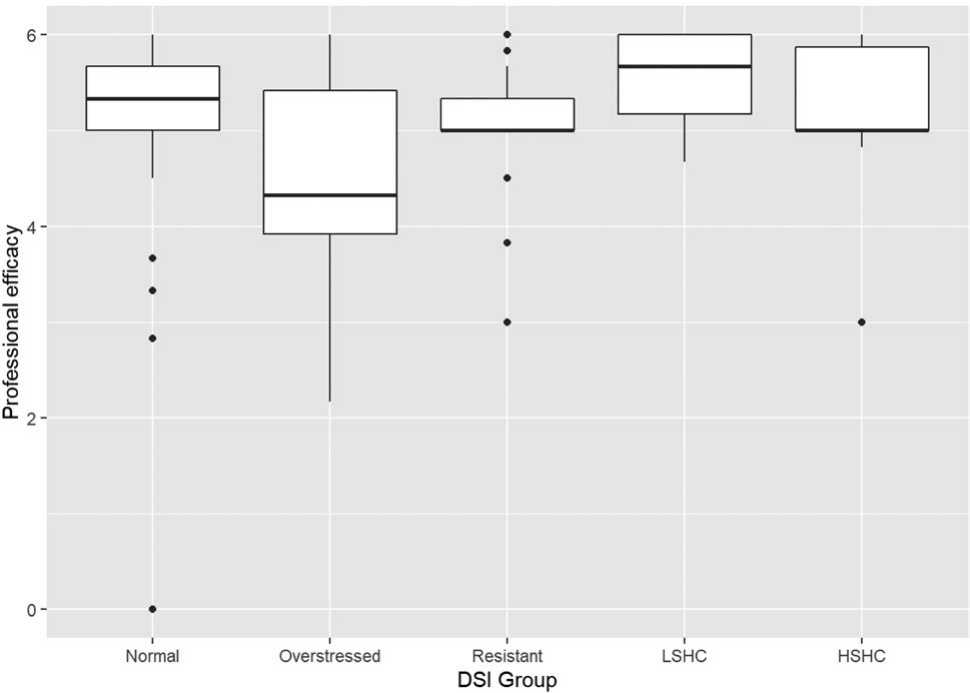
Boxplot of professional efficacy in the DSI groups. (*LSHC* low stress – high coping,* HSHC* high stress – high coping)

**Fig. 4:**
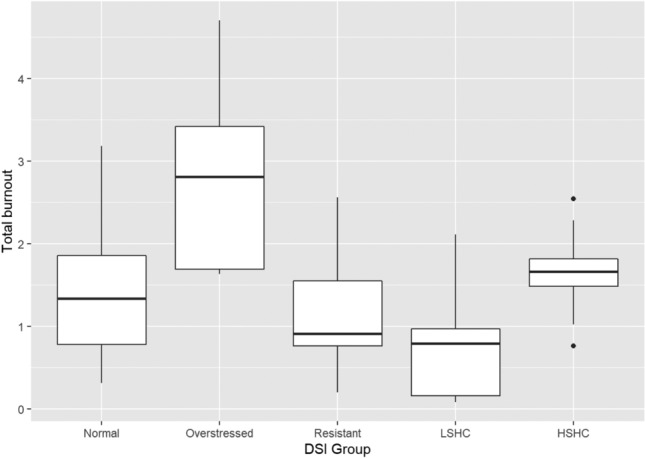
Boxplots of total burnout in the DSI groups. (*LSHC* low stress – high coping,* HSHC* high stress – high coping)

Levene’s test showed heteroskedasticity on three of the burnout variables, while the Q–Q plots showed no sign of departures from normality. Because of the heteroskedasticity, Welch’s *F* test was used to analyse group differences. These analyses showed significant differences between the DSI groups for emotional exhaustion  (F[4, 34] = 19.64, *p* < 0.001), cynicism (F[4, 34] = 6.47, *p* < 0.001), professional efficacy (F[4, 34]  = 4.04, *p* = 0.009), and total MBI (F[4, 34] = 17.29, *p* < 0.001). Groups differences are illustrated in Figs. 1, 2, 3, 4, and statistics are shown in Table 3.

**Table 3:** Descriptive statistics for Maslach burnout inventory scales

	Differential stress inventory groups						
	Total sample	Normal	Overstressed	Stress resistant	Low stress/high coping	High stress/high coping	DSI groups’ differences
	(*n* = 119)	(*n* = 58)	(*n* = 11)	(*n* = 21)	(*n* = 17)	(*n* = 12)	
Emotional exhaustion							
Mean ± SD	2.09 ± 1.29	2.16 ± 1.19	3.80 ± 1.32	1.52 ± 0.82	1.01 ± 0.80	2.77 ± 0.98	*F* = 19.64*,*
CI	(1.86, 2.33)	(1.85, 2.46)	(3.02, 4.58)	(1.17, 1.87)	(0.63, 1.39)	(2.21, 3.32)	*p* < .001
Median	1.8	2	3.8	1.4	1	3.1	–
Minimum	0	0	2	0.2	0.2	1.2	–
Maximum	5.8	4.8	5.8	3	3.2	4	–
Cynicism							
Mean ± SD	0.97 ± 1.02	0.91 ± 0.91	2.55 ± 1.42	0.69 ± 0.74	0.51 ± 0.57	1.00 ± 0.66	*F* = 6.47,
CI	(0.79, 1.16)	(0.68, 1.15)	(1.71, 3.38)	(0.37, 1.00)	(0.24, 0.78)	(0.63, 1.37)	*p* < .001
Median	0.6	0.6	2.6	0.6	0.4	0.8	–
Minimum	0	0	0.6	0	0	0	–
Maximum	4.6	4	4.6	2.4	1.8	2	–
Professional efficacy							
Mean ± SD	5.10 ± 0.90	5.11 ± 0.95	4.41 ± 1.23	5.04 ± 0.72	5.54 ± 0.47	5.19 ± 0.84	*F* = 4.04,
CI	(4.94, 5.26)	(4.87, 5.35)	(3.69, 5.13)	(4.73, 5.35)	(5.31, 5.76)	(4.72, 5.67)	*p* = .009
Median	5.17	5.33	4.33	5	5.67	5	–
Minimum	0	0	2.17	3	4.67	3	–
Maximum	6	6	6	6	6	6	–
MBI total							
Mean ± SD	1.40 ± 0.84	1.40 ± 0.68	2.76 ± 1.04	1.10 ± 0.62	0.69 ± 0.55	1.65 ± 0.49	*F* = 17.29,
CI	(1.25, 1.55)	(1.23, 1.58)	(2.14, 3.38)	(0.84, 1.37)	(0.44, 0.95)	(1.37, 1.92)	*p* < .001
Median	1.25	1.335	2.81	0.91	0.79	1.66	–
Minimum	0.08	0.31	1.63	0.2	0.08	0.76	–
Maximum	4.7	3.18	4.7	2.56	2.11	2.54	–

For continuous variables, mean, standard deviation, 95% confidence interval, median, minimum, and maximum values are reported, along with results from ANOVA. MBI total is calculated from Kalimo et al. (2003)

Games–Howell post hoc tests showed that, for emotional exhaustion, the overstressed group scored significantly higher than the normal group (*p* = 0.014), the stress-resistant group (*p* = 0.001), and the low stress–high coping group (*p* < 0.001). The high stress–high coping group scored higher than the stress-resistant group (*p* = 0.011) and the low stress–high coping group (*p* < 0.001), while the normal group scored higher than the low stress–high coping group (*p* < 0.001). On cynicism, the overstressed group scored significantly higher than all other groups (*p* < 0.05), while they did not differ significantly from each other. The Games–Howell post hoc test did not show any significant differences between the groups on the professional efficacy scale despite the omnibus test being significant. Finally, on the total burnout scale, the overstressed group scored significantly higher than all other groups (*p* < 0.05), while the low stress–high coping group scored significantly lower than the normal (*p* = 0.001), overstressed (*p* < 0.001), and high stress–high coping groups (*p* < 0.001).

### Analyses of HRV data

Levene’s test showed homoskedasticity in the DSI groups on all the continuous HRV variables. Q–Q plots showed no large departures from normality. Differences on the HRV variables in the DSI groups were examined using one-way ANOVA. There were no significant differences between any of the DSI groups on any of the HRV variables (*p* > 0.05). Fisher’s exact test showed no significant relationship between the DSI groups and normal or low stress zones (*p* = 0.873, only one participant deviated from those categories and was dropped from this specific analysis). Group differences are shown in Table 4.

**Table 4:** Descriptive statistics for heart rate variability variables

	Differential stress inventory groups						
	Total sample	Normal	Overstressed	Stress resistant	Low stress/high coping	High stress/high coping	DSI groups’ differences
	(*n* = 119)	(*n* = 58)	(*n* = 11)	(*n* = 21)	(*n* = 17)	(*n* = 12)	
Time domain							
SDNN (ms)							
Mean ± SD	60.74 ± 16.38	61.88 ± 15.35	54.17 ± 21.20	57.28 ± 14.47	63.14 ± 20.26	63.93 ± 13.28	*F* = 0.95,
CI	(57.80, 63.69)	(57.93, 65.83)	(41.64, 66.70)	(51.10, 63.47)	(53.51, 72.77)	(56.41, 71.44)	*p* = .437
Median	60,31	61,44	47,16	52,20	61,82	63,98	
Minimum	28,30	28,30	30,43	37,69	35,22	43,54	
Maximum	118,26	102,96	104,12	87,29	118,26	87,49	
RMSSD (ms)							
Mean ± SD	35.27 ± 15.53	37.54 ± 16.45	28.34 ± 13.44	31.54 ± 13.99	37.17 ± 15.92	34.49 ± 13.68	*F* = 1.24,
CI	(32.48, 38.06)	(33.30, 41.77)	(20.40, 36.28)	(25.55, 37.52)	(29.60, 44.73)	(26.75, 42.23)	*p* = .297
Median	31,97	35,40	23,15	31,34	30,76	33,36	
Minimum	10,97	10,97	14,60	15,78	13,61	14,74	
Maximum	82,75	82,75	52,36	69,23	69,29	63,87	
PNN50 (%)							
Mean ± SD	11.00 ± 9.27	12.18 ± 9.60	7.83 ± 9.37	7.87 ± 6.54	12.72 ± 10.93	11.20 ± 8.48	*F* = 1.32,
CI	(9.33, 12.66)	(9.71, 14.65)	(2.29, 13.37)	(5.08, 10.67)	(7.53, 17.91)	(6.41, 16.00)	*p* = .268
Median	9,44	10,02	3,39	8,89	8,66	11,38	
Minimum	0,19	0,19	0,58	1,16	0,28	0,67	
Maximum	41,47	41,47	26,41	20,77	36,68	29,54	
Frequency domain							
Total power (ms^2^)							
Mean ± SD	1,439.21 ± 953.56	1,510.71 ± 950.76	1,087.42 ± 822.14	1,238.51 ± 833.41	1,568.94 ± 1,143.54	1,583.51 ± 1,007.11	*F* = 0.83,
CI	(1,267.88, 1,610.53)	(1,266.03, 1,755.40)	(601.58, 1,573.26)	(882.06, 1,594.96)	(1,025.34, 2,112.53)	(1,013.69, 2,153.33)	*p* = .508
Median	1225,55	1236,46	719,24	907,89	1405,51	1421,77	
Minimum	235,93	235,93	286,84	355,02	381,89	522,81	
Maximum	4815,85	4815,85	2639,94	3084,05	4540,96	4195,91	
LF (n.u.)							
Mean ± SD	69.35 ± 11.69	68.06 ± 11.38	71.97 ± 7.49	72.58 ± 12.46	67.50 ± 14.93	70.18 ± 9.81	*F* = 0.83,
CI	(67.25, 71.45)	(65.13, 70.99)	(67.54, 76.40)	(67.26, 77.91)	(60.40, 74.59)	(64.63, 75.73)	*p* = .506
Median	71,96	71,70	70,59	77,59	72,66	69,55	
Minimum	31,36	36,28	58,14	46,04	31,36	54,04	
Maximum	88,32	84,91	83,19	88,32	86,55	85,10	
HF (n.u.)							
Mean ± SD	30.53 ± 11.64	31.81 ± 11.34	27.93 ± 7.45	27.30 ± 12.40	32.37 ± 14.83	29.74 ± 9.78	*F* = 0.83,
CI	(28.44, 32.62)	(28.90, 34.73)	(23.53, 32.33)	(22.00, 32.61)	(25.32, 39.42)	(24.21, 35.27)	*p* = .507
Median	27,95	28,12	29,27	22,33	27,27	30,32	
Minimum	11,64	15,06	16,78	11,64	13,42	14,86	
Maximum	68,19	63,61	41,64	53,84	68,19	45,91	
LF/HF ratio							
Mean ± SD	2.75 ± 1.40	2.53 ± 1.22	2.85 ± 1.16	3.33 ± 1.71	2.74 ± 1.68	2.74 ± 1.34	*F* = 1.28,
CI	(2.50, 3.01)	(2.22, 2.85)	(2.16, 3.53)	(2.60, 4.07)	(1.94, 3.54)	(1.98, 3.50)	*p* = .283
Median	2,57	2,55	2,41	3,48	2,66	2,30	
Minimum	0,46	0,57	1,40	0,85	0,46	1,18	
Maximum	7,59	5,64	4,96	7,59	6,45	5,73	
LF peak (Hz)							
Mean ± SD	0.06 ± 0.02	0.06 ± 0.02	0.05 ± 0.01	0.06 ± 0.02	0.06 ± 0.02	0.06 ± 0.03	*F* = 1.04,
CI	(0.06, 0.06)	(0.05, 0.06)	(0.05, 0.06)	(0.05, 0.07)	(0.06, 0.07)	(0.05, 0.08)	*p* = .389
Median	0,05	0,05	0,05	0,05	0,06	0,05	
Minimum	0,04	0,04	0,04	0,04	0,04	0,04	
Maximum	0,12	0,10	0,08	0,10	0,09	0,12	
HF peak (Hz)							
Mean ± SD	0.20 ± 0.06	0.21 ± 0.07	0.20 ± 0.06	0.18 ± 0.05	0.19 ± 0.06	0.19 ± 0.06	*F* = 1.29,
CI	(0.19, 0.21)	(0.19, 0.23)	(0.17, 0.24)	(0.16, 0.20)	(0.16, 0.22)	(0.16, 0.22)	*p* = .278
Median	0,15	0,20	0,19	0,15	0,15	0,15	
Minimum	0,15	0,15	0,15	0,15	0,15	0,15	
Maximum	0,35	0,35	0,32	0,31	0,32	0,30	
Non-linear							
SD1 (ms)							
Mean ± SD	24.94 ± 10.98	26.54 ± 11.63	20.04 ± 9.50	22.30 ± 9.89	26.28 ± 11.25	24.39 ± 9.67	*F* = 1.24,
CI	(22.97, 26.91)	(23.55, 29.54)	(14.43, 25.65)	(18.07, 26.53)	(20.93, 31.63)	(18.91, 29.86)	*p* = .297
Median	22,61	25,03	16,37	22,16	21,75	23,59	
Minimum	7,76	7,76	10,32	11,16	9,62	10,43	
Maximum	58,52	58,52	37,02	48,95	49,00	45,16	
SD2 (ms)							
Mean ± SD	47.44 ± 14.26	48.52 ± 13.61	41.16 ± 13.89	44.66 ± 14.98	49.20 ± 15.79	50.29 ± 14.14	*F* = 1.00,
CI	(44.87, 50.00)	(45.02, 52.03)	(32.95, 49.37)	(38.25, 51.07)	(41.69, 56.70)	(42.29, 58.29)	*p* = .410
Median	45,91	46,32	35,88	39,07	47,07	48,02	
Minimum	21,19	21,19	22,99	24,72	24,07	31,77	
Maximum	81,44	81,44	65,73	74,22	80,47	81,31	
*α*_1_							
Mean ± SD	1.20 ± 0.16	1.18 ± 0.14	1.24 ± 0.14	1.25 ± 0.17	1.17 ± 0.23	1.22 ± 0.15	*F* = 0.99,
CI	(1.17, 1.23)	(1.14, 1.22)	(1.16, 1.32)	(1.17, 1.32)	(1.06, 1.28)	(1.13, 1.30)	*p* = .418
Median	1,23	1,21	1,23	1,30	1,24	1,20	
Minimum	0,66	0,77	0,98	0,80	0,66	0,92	
Maximum	1,53	1,48	1,48	1,49	1,53	1,46	
*α*_2_							
Mean ± SD	0.49 ± 0.06	0.49 ± 0.06	0.50 ± 0.07	0.50 ± 0.06	0.48 ± 0.07	0.49 ± 0.08	*F* = 0.26,
CI	(0.48, 0.50)	(0.47, 0.50)	(0.46, 0.54)	(0.47, 0.52)	(0.45, 0.51)	(0.44, 0.54)	*p* = .906
Median	0,49	0,49	0,49	0,50	0,47	0,46	
Minimum	0,36	0,36	0,41	0,36	0,36	0,38	
Maximum	0,66	0,65	0,64	0,60	0,62	0,66	
Indices							
PNS index							
Mean ± SD	− 0.76 ± 0.74	− 0.66 ± 0.74	− 0.96 ± 0.86	− 0.88 ± 0.56	− 0.76 ± 0.89	-0.82 ± 0.75	*F* = 0.61,
CI	(− 0.89, − 0.62)	(− 0.85, − 0.47)	(− 1.47, − 0.45)	(− 1.12, − 0.64)	(− 1.19, − 0.34)	(− 1.24, -0.39)	*p* = .658
Median	− 0,85	− 0,79	− 1,22	− 0,99	− 0,96	− 0,65	–
Minimum	− 2,28	− 2,28	− 2,19	− 2,04	− 1,96	− 1,95	–
Maximum	1, 58	1, 47	0,81	0,13	1,58	0,15	–
SNS index							
Mean ± SD	0.43 ± 0.79	0.35 ± 0.76	0.60 ± 1.02	0.47 ± 0.66	0.52 ± 0.86	0.46 ± 0.87	*F* = 0.34,
CI	(0.29, 0.57)	(0.16, 0.55)	(− 0.00, 1.20)	(0.19, 0.76)	(0.11, 0.93)	(− 0.03, 0.95)	*p* = .853
Median	0,40	0,31	0,68	0,41	0,67	0,18	–
Minimum	− 1,62	− 1,43	− 1,16	− 0,75	− 1,62	− 0,41	–
Maximum	2, 56	2, 56	2, 47	2, 13	1, 91	2, 21	–
Stress index							
Mean ± SD	7.75 ± 1.79	7.54 ± 1.74	8.56 ± 2.26	7.98 ± 1.66	7.72 ± 1.82	7.65 ± 1.80	*F* = 0.85,
CI	(7.43, 8.07)	(7.09, 7.99)	(7.22, 9.90)	(7.27, 8.69)	(6.86, 8.59)	(6.63, 8.67)	*p* = .497
Median	7, 77	7,59	9, 16	8, 15	8, 09	7, 22	–
Minimum	3, 86	3, 86	5, 24	5, 03	3, 86	4, 91	–
Maximum	12, 39	12, 39	12, 16	11, 41	11, 64	10, 95	–
Stress zones							
Low	46 (38.7%)	24 (41.4%)	4 (36.4%)	6 (28.6%)	7 (41.2%)	5 (41.7%)	*p* = .873
Normal	72 (60.5%)	33 (56.9%)	7 (63.6%)	15 (71.4%)	10 (58.8%)	7 (58.3%)	–
Elevated	1 (0.8%)	1 (1.7%)	0 (0.0%)	0 (0.0%)	0 (0.0%)	0 (0.0%)	–

For continuous variables, mean, standard deviation, 95% confidence interval, median, minimum, and maximum values are reported, along with results from ANOVA. For categorical variables, n and % are reported, along with results from Fisher's exact test

*LF*  low frequency, *HF*  high frequency, *n.u.*  normalised units, *SDNN* standard deviation of RR intervals, *RMSSD*  square root of the mean squared differences between successive RR intervals. *PNN50*  % of successive RR interval pairs that differ more than 50 ms. *α*_1_   short-term fluctuations of detrended fluctuation analysis, *α*_2_  long-term fluctuations of detrended fluctuation analysis, *PNS*  parasympathetic nervous system, *SNS*  sympathetic nervous system, *Stress index*  square root of Baevsky’s stress index. Heart rate variability was recorded over 24 h

## Discussion

This publication presents novel data for the up-to-now rarely investigated Differential Stress Inventory and its subjective and objective recorded overload. There were group differences on the burnout dimensions emotional exhaustion and cynicism between DSI types. Also, on the total burnout scale, the overstressed type scored significantly higher than all other groups. These group differences were not present with regards to the more objective measures of heart rate variability. There were no significant differences between the DSI types on gender or workgroup, and the groups were found to be not equal with respect to age, height, and weight.

Work overload is a widespread problem nowadays and is associated with mental illness and burnout (Beer et al. 2016). The overstressed type suffers significantly more than average from stress caused by unavoidable everyday activities, according to the DSI (Lefèvre and Kubinger 2004), and this was reflected in our data as the overstressed type had the most adverse scores on all the MBI scales. The high stress/high coping (HSHC) type scored higher on emotional exhaustion than the stress-resistant type and the low stress/high coping (LSHC) type. The HSHC type also scored higher than the LSHC type on total burnout. It is unsurprising that the HSHC group scored higher than the stress-resistant group, as the latter experienced less stress. It is more interesting that the HSHC type scored higher than the LSHC type, because it indicates that the HSHC type is more burdened than the other types despite feeling like they cope well with the stress. The Normal type showed higher values for emotional exhaustion than for the stress-resistant and LSHC types. Personal details were not asked of the participants, and we could thus not control for life events, or taking care of relatives, which is a limitation of the study. The health care situation at home is also associated with a lower health balance. One in four people who are burdened by caring for relatives also reported that they have had mental illnesses such as burnout, depression, or anxiety (Hillienhof 2013).

The overstressed type had the highest level of cynicism. A systematic review showed that cynicism was associated with workplace justice, demands, high work load, low reward, low supervisor support, low co-worker support, and job insecurity (Aronsson et al. 2017 Mar 16). The HSHC type had the second highest values on cynicism. Considering the HSHC type’s high level of emotional exhaustion and total burnout, this seems alarming. High stress and high coping will probably only be successful as long as there is social support at work and home. In a study of students, social support was associated with resilience to stress (Park et al. 2015). In contrast, low social support outside the workplace was associated with more burnout (Boland et al. 2019). Resilience and social support were not investigated in this study, which is a limitation. Future studies could measure social support to see if it moderates the level of burnout in the HSHC types. An international literature search revealed no relevant studies on DSI and burnout, so the available data must be considered new. Therefore, we discuss other studies that used different stress-related instruments. A study confirmed the independent roles of effort reward imbalance and intrinsic overcommitment for mental disorders. There are stronger associations among women (Lidwall 2016). Our results failed to replicate this gender difference. Risk patterns of work-related behavioural and experience (AVEM) are also associated with effort–reward imbalance, chronic stress, and reduced mental health (Voltmer et al. 2017).

In occupational medicine, heart rate variability is seen as a physiological stress indicator (Böckelmann and Seibt 2011; The et al. 2020). Taking into account the five different DSI types, we found no significant differences in any of the physiological stress parameters. Searches for “differential stress inventory” yielded no results on Google Scholar or PubMed. We did find only one study by Gaurav et al. (2018) in which the authors trained an algorithm to predict with better than chance results, based on EEG data, whether participants belonged to a high stress (DSI types overstressed or HSHC) or low stress (DSI types normal, stress resistant, or LSHC) conditions. We found no studies regarding DSI and HRV in PubMed, Google Scholar, or PsycINFO.

It is interesting that we found differences in MBI scales but not in HRV parameters. This could be caused by common method variance. The common method variance could mean that people who felt that they were stressed tended to answer in a way that gave them high scores on both DSI and MBI scales. Another potential reason for why we found differences in MBI scales and not HRV parameters might be that DSI and MBI measure more long-term stress and stress consequences, while HRV measures short-term stress. Participants might have had a particularly un-stressful day the 24 h they were measured, though they might still be aware that they are stressed most days. This is evidenced by all but one participant being placed in stress zones normal and low. Other studies have shown the relationship between stress, burnout, and reduced HRV (Togo and Takahashi 2009; Lennartsson et al. 2016; Lo et al. 2020; The et al. 2020). Studies also showed the dependence of HRV on different coping styles (Fuller 1992; Laborde et al. 2015). A study demonstrated gender-specific differences and correlations with age for HRV parameters and coping mechanisms. In younger men (age 18–30 years), a higher active coping was associated with less global autonomic activity or SDANN. In young men, expression of negative emotions or anger was associated with LF power, but the same was not found for elderly men (Ramaekers et al. 1998). Persons with more submissive behaviour and higher perception of psychophysiological arousal showed a higher sympathetic dominance in HRV (Sgoifo et al. 2003).

The results of this study are only partially transferable, as female subjects are overrepresented. In this study, 85% of the total sample were female, while only 46.5% of those in employment in Germany are female (Federal Institute for Population Research 2018). However, the work groups that we sampled are traditionally overrepresented by women, and so, it can be considered transferable to those occupational groups. As we collected the data in local bank and not in headquarters of banks or institutions, we met more women (Bundesagentur für Arbeit 2019). There was an activity protocol for the ECG and HRV evaluation. The subjects were instructed to investigate everyday stresses and strains. The filling out of the protocols was dependent on the subjects and was handled differently. It should be checked whether separate analyses of heart rate variability for working time, leisure time, and sleeping time are reasonable. This is particularly useful in the case of noticeable subjective information in the MBI, e.g., the question of an existing recovery phase during sleep. The working conditions of both workgroups are similar in terms of working hours and lack of shift work. Because of their activities on workplace, the medical assistance may be more active than bank employees. The degree of movement during work was not recorded.

## Conclusions

We found differences in burnout scores among the DSI types, which indicate that knowledge about DSI types could be used to identify people at risk of burnout. This is important, because burnout can have adverse health consequences for employees. This could also be seen as support for the validity of the differential stress inventory. However, we found no differences among the DSI types on any of the HRV parameters. This can indicate that the DSI types do not capture any of the short-term or physical stress the body experiences, only long-term or mental stress. It could also be interpreted against the validity of the DSI. The DSI thus may have some validity as a measurement of long-term stress, subjective experience of stress, or stress phenomena related to burnout, but not at short-term, physical stress. These comparisons could moreover be used for building hypotheses for further studies aiming at clarifying relations between DSI groups and burnout. Categorisations like in the DSI can be helpful to non-experts, because it offers an easy and intuitive understanding of complex phenomena like stress, coping, and burnout. As such, it can be favourably applied in the occupational setting. Occupational health consultants are in the unique position that they may discover cases of burnout before they become a clinical problem that is then handled by a general practitioner. They may thus benefit from using the DSI in cooperation with the employees to help assess their own situation and initiate targeted preventive measures before they reach into clinical pathology.
